# Developing a comprehensive abortion prevention program for couples based on I-change model: study protocol for a mixed method research

**DOI:** 10.1186/s12978-024-01816-y

**Published:** 2024-06-07

**Authors:** Zari Dolatabadi, Maryam Moridi, Farnaz Farnam, Maryam Damghanian

**Affiliations:** 1 Reproductive Health and Midwifery Department, School of Nursing and Midwifery, Tehran University of Medical Sciences, Tehran, Iran; 2 Nursing and Midwifery School, Student Research Committee, Tehran University of Medical Sciences, Tehran, Iran; 3 Nursing and Midwifery Care Research Center, Midwifery and Reproductive Health Department, School of Nursing and Midwifery, Tehran University of Medical Sciences, Tehran, Iran; 4 Department of Reproductive Health, School of Nursing and Midwifery, Tehran University of Medical Sciences, Tehran, Iran

**Keywords:** Abortion, Induced abortion, Illegal abortion, Pregnancy termination, I-change model, Decision-making, Prevention, Influencing factors, Determinant factors

## Abstract

**Background:**

In countries where abortion laws are stringent, induced abortions are prevalent. The limited availability of abortion services within these regions amplifies the likelihood of maternal complications and mortality. Induced abortions represent a significant public health concern in Iran and are characterized by a multitude of intricate factors that remain largely unexplored. Gaps in knowledge persist pertaining to the influences driving induced abortion within the Iranian context. To adequately address the issue of induced abortion, it is imperative to discern the determinants that shape the decision-making process. The primary objective of this study was to design an intervention program focused on mitigating the occurrence of induced abortion within couples, with an emphasis on identifying the key factors that contribute to this phenomenon.

**Methods:**

This study comprises three phases. In the first phase, a qualitative approach based on the I-change model will be employed to identify the factors influencing induced abortion. The second phase involves a systematic review to identify the determinants of induced abortion and strategies to prevent induced abortion. In the third phase, the outcomes of the qualitative approach and systematic review will be shared with experts and specialists using the Delphi method to categorize and prioritize strategies. Subsequently, based on the final consensus, a comprehensive program will be developed to prevent induced abortion.

**Discussion:**

This study introduces an I-change model-based program for the prevention of induced abortion. The prevention of induced abortion holds great significance in mitigating maternal morbidity and mortality, curtailing healthcare expenses, and fostering population growth rates. The research findings will be disseminated via reputable peer-reviewed journals and communicated to the academic and medical communities. This dissemination aims to provide valuable insights that can contribute to the advancement of induced abortion and abortion prevention programs.

## Abstract (plain language summary)

Induced abortion is restricted in Iran, and many married women with unintended pregnancies resort to clandestine procedures, resulting in adverse health outcomes. Induced abortion contributes to 2.3% of maternal mortality rates in the country. Understanding the factors influencing abortion is crucial for designing effective preventive interventions. Due to cultural and political sensitivities, reliable data on the determinants of induced abortion in Iran, particularly from the perspective of male partners, remain scarce. Existing research primarily focuses on women’s views, neglecting the potential influence of men on abortion-related decisions. This study aims to bridge this gap by investigating the determinants of induced abortion in couples and developing a comprehensive couple-based abortion prevention program in Iran. Employing a mixed-methods approach, this study explored the factors associated with couples’ decisions regarding abortion. Subsequently, a systematic review will identify existing knowledge on abortion determinants and preventive strategies. On the basis of this comprehensive understanding, an evidence-informed abortion prevention plan will be devised. The findings of this study can inform policymakers and population/reproductive health experts, ultimately aiming to reduce the burden of negative health and social consequences associated with abortion, leading to cost reductions and improved health outcomes for women, families, and society.

## Background

Induced abortion is characterized by the deliberate termination of pregnancy and represents a prevalent reproductive phenomenon, with an annual occurrence exceeding 73 million instances [1]. During the period from 2015 to 2019, it is estimated that approximately 48% (equivalent to 121 million) of all pregnancies globally were unintended. Among these occurrences, induced abortion constituted 61% (equivalent to 73 million) of the cases [2]. In Iran, accurate statistics on abortion are not available due to the sensitive nature of the issue. Nevertheless, there has been a noticeable increase in abortion rates in Iran over the last decade [3].

In Iran, the regulations pertaining to induced abortion are distinguished by stringent guidelines and restricted circumstances under which the procedure is allowed. The general stance is that induced abortion is prohibited unless specific conditions are met. These conditions include situations where there is a substantial threat to the mother’s life or when the fetus has been diagnosed with a recognized disease or defect that is considered an exception by the Legal Medical Organization of the country [4]. The exception for cases involving a threat to the woman’s life recognizes the importance of safeguarding maternal health and prioritizing life preservation in critical situations. It allows for the termination of the pregnancy when necessary to protect the woman from severe harm or potential loss of life. Another exception applies to cases where the fetus has been diagnosed with a recognized disease or defect. The Legal Medical Organization of Iran evaluates individual cases to determine whether the diagnosed condition qualifies as an exception for induced abortion. This approach acknowledges the complexities of certain medical circumstances and permits the termination of pregnancies when the fetus’s health or quality of life is significantly compromised [5]. If these conditions are not met, women seeking to terminate their unwanted pregnancies have no alternative but to resort to clandestine and potentially hazardous abortion methods [4].

Unsafe abortion is a significant factor contributing to maternal mortality globally [6]. However, there is limited information available regarding the extent of maternal mortality and the associated health risks related to unsafe abortions in Iran. A study conducted by Zalvand et al. emphasized the role of abortion as a direct contributor to maternal mortality in Iran [7]. Moreover, according to an indirect estimation, approximately 5% of maternal fatalities associated with pregnancy can be ascribed to complications associated with postabortion interventions [8].

The literature documented the various consequences of unsafe abortion, including physical complications such as sepsis, hemorrhage, and genital trauma [9]. The repercussions of induced abortion extend beyond individual consequences and have wider ramifications for the healthcare system. Addressing these complications necessitates substantial resource allocation, encompassing hospital beds, blood supply, medications, and other essential healthcare services [10]. Furthermore, induced abortion also has implications for the demographic situation of the country; despite Iran’s population experiencing an overall increase based on the most recent census conducted in 2016, there has been a decrease in the total fertility rate (TFR) coupled with an increase in life expectancy, leading to rapid growth of the elderly population. Notably, that Iran is undergoing an aging process at a faster pace than other nations [11, 12]. The decision to undergo induced abortion is influenced by a range of factors that are often unique to individuals and can vary significantly. Individual factors encompass aspects such as marital status, experiences of sexual assault or familial abuse, financial self-sufficiency, and level of educational achievement. Interpersonal factors include considerations of the partner’s circumstances and the availability of parental support. Societal determinants include social norms, religious beliefs, stigmatization related to premarital and extramarital sexual activity, adolescent status, and societal autonomy. Organizational factors include the quality and accessibility of sex education, the healthcare system, and the legal framework surrounding abortion [13–16].

Although the determination to either terminate or proceed with an unintended pregnancy is commonly regarded as a personal issue, the involvement of spouses in reproductive behaviors and decision-making plays a pivotal role [17]. Men play a key role in matters related to sexual and reproductive health and rights (SRHR) [18]. Research has shown that involving men in reproductive health initiatives is associated with a reduced likelihood of unsafe abortions [19]. Previous studies on decision-making regarding pregnancy termination have focused primarily on women’s preferences and perspectives. However, it is important to recognize that decisions concerning pregnancy are often influenced by the male partner, indicating the involvement of distinct fertility motives [20].

To develop effective programs aimed at preventing and reducing induced abortion, it is crucial to gain a deeper understanding of this phenomenon and its underlying factors. Therefore, this study will utilize the I-change model as an integrated framework to enhance our comprehensive understanding of induced abortion and its determinants within couples. The I-Change model is built upon the foundational framework of the Attitude Social Influence-Self-efficacy (ASE) model, which shares similarities with the Theory of Planned Behavior [21]. Nevertheless, the I-Change model incorporates supplementary components, including modeling and social support as influential factors, in addition to subjective norms. By amalgamating the ASE model with the understandings derived from stages of change models and action planning models, the I-change model presents a comprehensive framework for scrutinizing and fostering processes of behavior change [22–25].

The process of behavioral change can be categorized into three distinct phases: premotivational, motivational, and postmotivational. Each phase is characterized by specific determinants that contribute to the overall change process. During the premotivational phase, the key focus lies in creating awareness, where factors such as behavioral cognizance (consciousness of one’s own behavior), knowledge (comprehension of the behavior and the associated information that stimulates action), risk perception (evaluation of the probability and severity of a health threat), and cues to action (internal or external prompts that initiate behavior) play significant roles. The motivational phase comprises several key elements, including attitude, social influence, self-efficacy, and intention. Attitude involves the assessment of the benefits and drawbacks associated with a particular behavior, considering its positive and negative aspects. Social influence encompasses the consideration of societal norms and the opinions of individuals closely connected to the behavior, as well as the observation of others who engage in behavior and the response to social pressures exerted by the surrounding environment. Self-efficacy pertains to an individual’s belief in her or his own capability to successfully perform or refrain from the behavior in question. Finally, intention denotes the conscious decision and commitment to engage in or avoid behavior based on personal goals and motivations. The postmotivational or action phase represents the stage that is most closely associated with the actual execution of the behavior. This phase encompasses various activities, including the formulation of action plans, the implementation of these plans (plan enactment), and the resolution of personal skills or barriers that may hinder the desired action [26].

The cultural and social contexts in Iran pose distinctive challenges and necessitate careful consideration in regard to the topic of abortion. The attitudes and practices surrounding abortion in the country are significantly influenced by religious and legal factors.

This study acknowledges the importance of bridging the gap between the theoretical knowledge and lived experiences of couples. By exploring the real experiences of couples, this study aimed to identify the significant factors that influence decision-making regarding abortion. It seeks to move beyond theoretical frameworks and gain insights from individuals who face complex choices in their reproductive lives. This approach allows for a deeper understanding of the contextual nuances and challenges that couples encounter when making reproductive decisions. The ultimate objective of this research is to utilize the knowledge gained to develop a program aimed at preventing and reducing abortion.

## Methods

### Objectives

The overall objective of this study is to develop an abortion prevention program based on the I change model, with an emphasis on identifying the key factors that contribute to induced abortion.

### Study design

This study employs a mixed methods research design consisting of three distinct phases. The first phase involves conducting semistructured interviews using a qualitative approach guided by the I-Change model. The primary objective of this research is to gain a comprehensive understanding of the factors that influence couples’ intentions to seek induced abortion. These interviews provide in-depth insights into the determinants and considerations involved in the decision-making process.

In the second phase, a systematic review will be conducted to identify the determinants of induced abortion and examine existing models and strategies implemented to prevent it. This review aims to provide a comprehensive overview of the various approaches and interventions employed in similar contexts. The findings from the first phase, which highlight the factors identified through the interviews, will be compared and aligned with the strategies identified in the reviewed studies. This process allows for the selection of the most relevant and effective strategies to address the identified factors.

In the final phase, the Delphi method will be employed to establish a rational and well-suited framework for a comprehensive program. This method involves gathering input and feedback from a panel of experts in the field. The experts will quantify the main strategies, criteria, and alternative options for the program, ensuring a systematic and rigorous approach. This process also facilitates the integration of emerging strategies with the overarching goal of the program, promoting a cohesive and effective approach to preventing induced abortion.

Overall, this study utilizes a mixed methods design, incorporating qualitative interviews, systematic review, and the Delphi method. Through these phases, the research aims to gain a comprehensive understanding of the factors influencing couples’ intentions regarding induced abortion, identify effective prevention strategies from the literature, and develop a well-structured and evidence-based program.

### Outcome measures

The primary objective of this study is to investigate the factors influencing couples’ decision-making processes regarding abortion. The central research question guiding this inquiry is: “What are the determinants that influence couples’ choices related to abortion?” To address this primary question, the study aims to achieve the following key outcomes:

1. Identify the determining factors of induced abortion from women’s perspectives, based on the I-Change model.
2. Identify the determining factors of induced abortion from men’s perspectives, based on the I-Change model.
3. Examine the determining factors of induced abortion from the experts’ point of view, using the I-Change model.
4. Identify the determining factors of induced abortion through a systematic review.
5. Explore the strategies employed to prevent induced abortion in the reviewed studies.
6. Identify the components and strategies of a comprehensive program for preventing induced abortion.

By addressing these outcomes, this study aims to gain insights into the factors influencing couples’ decision-making processes regarding abortion and develop a comprehensive program to prevent induced abortion effectively.

### Study phases

This mixed-method study will consist of three distinct phases, outlined as follows:

#### Phase 1 - qualitative research: understanding the factors influencing couples’ intentions and behaviors related to induced abortion

​​​​​​​In this initial phase, a directed content analysis study will be conducted based on the I-Change model. Cases of induced abortion registered in the electronic databases of primary health centers (PHCs) and hospitals will be selected, adhering to specific inclusion criteria. The study will include women aged 15–45 years who have had an induced abortion within the past year, with no history of medical indications for abortion, as well as their spouses. The researchers will establish communication with a targeted sample of couples and provide a comprehensive explanation of the study’s objectives. Informed consent will be obtained from participating couples after ensuring their full understanding of the research’s purpose and nature. Face-to-face interviews with couples interested in participating in the study will then be scheduled at a suitable place and time.

Data collection in this study will primarily involve conducting semi-structured interviews administered by the first author of the research. Interview questions will be developed based on the constructs of the I-Change model, expert panel opinions, and a preliminary study. The interviews will begin with a framework question about the couples’ emotions and perceptions upon discovering the pregnancy for the first time. Subsequently, more general questions will be asked about the couples’ perceptions, attitudes, and behaviors leading to abortion. Couples will be interviewed together or individually based on their preferences in a private, quiet, and comfortable setting. Additionally, individual interviews will be conducted with experts and policymakers in the field of reproductive and population health to apply a triangulation approach and gain a comprehensive understanding of feasible strategies to prevent induced abortion.

The qualitative data will be analyzed using the directed qualitative content analysis method [27]. Primary categories for examination will be derived from the framework provided by the I-Change model, serving as the structure for organizing and understanding the data. With participants’ consent, the interviews will be digitally recorded and transcribed verbatim. Each transcript will be assigned a distinct code to ensure confidentiality. The audio files and transcripts will be reviewed multiple times to fully engage with the data and address any potential ambiguities or inconsistencies. A summary of the key issues discussed in each interview will be shared with participants for member checking to ensure accurate interpretation of their comments. Data analysis will be conducted concurrently with data collection, treating all words, sentences, and paragraphs as interconnected components. Codes will be derived, leading to the formation of subcategories and eventually main categories. The categories will be abstracted and integrated into the I-Change model structures. MAXQDA software will be used for data management. The sample size for interviews will be determined based on information saturation, expanding the sample until no new themes emerge from participants’ input. Selection criteria will consider participants’ age, occupation, educational status, and economic status to ensure maximum variation.

#### Phase 2 - systematic review study: determinants of induced abortion

To identify and explore the determinants of induced abortion and strategies to prevent it, a systematic review of qualitative and quantitative studies will be conducted. This phase of the study will follow the guidelines provided by the “Preferred Reporting Items for Systematic Reviews and Meta-Analysis (PRISMA)” checklist for systematic reviews [28]. The PRISMA guidelines consist of 27 items that cover different aspects of a systematic review, including abstracts, methods, results, discussions, and financial resources.

### Search strategy

A comprehensive and systematic search of English language articles will be conducted across multiple databases, including PubMed, Web of Science, Medline, Embase, Scopus, and Cochrane Library. Furthermore, the reference lists of the retrieved articles will be manually reviewed to identify relevant studies. The search will encompass articles published between 2000 and 2024. Initially, keywords relevant to the study objectives were selected, and the following search strategy was employed:

1. “abortion” OR “pregnancy termination”.
2. “induced” OR “septic” OR “illegal” OR “drug-induced” OR “criminal” OR “intentional” OR “unsafe”.
3. “determinants” OR “causes” OR “factors” OR “influencing factors” OR “perception” OR “behaviors” OR “decision-making”.
4. “prevention” OR “preventive strategies”.
5. #1 AND #2 AND #3 AND #4 AND

### Eligibility screening

To be included in the review, studies must meet the following criteria: they should be published in peer-reviewed journals with accessible full-text articles and have transparent findings. The eligible studies can be qualitative, quantitative, or mixed-method. Studies will be excluded for the following reasons: (1) not published in English; (2) categorized as editorials, letters to the editor, commentaries, viewpoint pieces, policy briefs, newspapers, newsletters, and conference summaries; and (3) focused specifically on spontaneous abortion or the medical causes and consequences of abortion.

### Study selection

The process of searching and reviewing articles will be conducted collaboratively by two members of the research team. Two researchers will independently review the titles and abstracts of the articles to select relevant studies. Subsequently, the full texts of the selected studies will undergo a thorough examination. Each reviewer will independently extract data from each study using a standardized template. The extracted information will include details such as study authors, study objectives, study setting (including location(s) and year(s) of publication), inclusion and exclusion criteria, participant characteristics, study design, sample size, data collection methods, analytical approaches, results, strengths and limitations, and any other relevant information that is deemed necessary.

### Quality appraisal

The methodological quality of the included studies will be assessed using the Mixed Methods Appraisal Tool (MMAT), a reliable and validated tool specifically designed for systematic mixed studies reviews [29]. The MMAT includes criteria for evaluating qualitative studies as well as three types of quantitative study designs: randomized controlled trials, nonrandomized studies, and descriptive studies. Each study will be assessed based on five criteria according to the provided guidelines. Studies that meet only two criteria will be considered low quality and excluded from the review. Studies that meet three criteria will be classified as medium quality, while those meeting four or five criteria will be considered higher quality. Only studies meeting the quality criteria will be included in the review. The assessment of methodological quality will be performed independently by two reviewers (ZD and MM), with any disagreements resolved through consensus. If consensus cannot be reached, the input of another reviewer (MD) will be sought. The results of the quality assessment will be integrated into the synthesis process in a narrative manner.

#### Phase 3: development of an abortion prevention program

The objective of this phase is to develop a comprehensive and practical program aimed at preventing induced abortion. To design an effective abortion prevention program, we will begin by conducting qualitative and systematic review studies to investigate the factors that influence induced abortion and identify potential preventive strategies (phases 1 and 2). Subsequently, the identified strategies will be evaluated in light of the study findings to provide insights for program development. Interventions and programs will be reviewed, and consensus methods will be employed to further assess and prioritize them.

The Delphi method, widely used in medical and health research, will be employed as a consensus approach. This method facilitates decision-making processes, particularly in situations where evidence is limited, unclear, or when the perspectives of key stakeholders may differ. The Delphi method follows a systematic process involving survey rounds, information gathering, and group consensus, aiding in the prediction and decision-making process [30]. To achieve our objective, we will utilize the Delphi method, a reliable approach for collecting and integrating expert opinions. This method has been extensively used in medical research to generate recommendations and has proven effective in addressing various topics such as barriers in clinical research, research priorities, and educational needs. Its successful application in similar studies [31] further justifies its adoption for this investigation.

The panel of experts for this study will comprise specialists in reproductive health, midwifery, gynecology, forensic medicine, population policy, and healthcare policy. These experts will be selected using purpose-based sampling methods. The number of participants in this section will range from 10 to 15 experts.

The proposed programs, based on the results of the previous two phases, will be presented to the specialists. Subsequently, the opinions of the experts regarding the prioritization of appropriate methods at each phase will be assessed. The results of the Delphi rounds will be collected and analyzed. The number of Delphi rounds will be repeated as necessary to reach a saturation of opinions. The findings from the Delphi rounds will be presented to the expert panel, where the abortion prevention program will be finalized.

## Discussion

The objective of this study is to develop a program aimed at preventing induced abortion among Iranian couples. Despite the existence of strict abortion laws, the occurrence of induced abortions has been on the rise in recent years. In order to address this issue and achieve population goals, particularly among the younger demographic, which seeks to elevate the Total Fertility Rate (TFR) above replacement level and enhance key indicators in alignment with broader population policies, it is crucial to place significant emphasis on thorough monitoring of abortion rates nationwide. This monitoring involves summarizing reports from relevant institutions and conducting related research in the field. In order to address this phenomenon and its determining factors more effectively, it is necessary to design interventions aimed at preventing and reducing induced abortion. The decision to pursue an abortion is influenced by various contextual factors that can fluctuate over time [32]. The decision-making process is intricate, iterative, and influenced by the socioeconomic and power dynamics that exist between women and their significant others [15, 33]. The decision-making process occurs within a context characterized by uncertainty, where conflicting rationalizations and emotional experiences come into play. It is crucial to acknowledge that the decision-making process regarding abortion is not isolated; rather, it is influenced by various factors. These factors include women’s autonomy, the involvement of their partners, family members, and social networks, and the broader abortion landscape, encompassing relevant laws, policies, and elements of the healthcare system [34, 35]. Research indicates that decision-making processes concerning abortion exhibit similar patterns in both liberal and restrictive environments. Notably, abortions occur even in countries with stringent laws. Hence, the reduction of abortion rates cannot be solely accomplished by imposing restrictive legislation. To achieve tangible results, it is imperative to develop effective interventions and programs that are founded upon a comprehensive understanding of the factors contributing to abortions, as well as the unique requirements of the target populations.

This study exhibits several significant strengths that bolster its robustness and the reliability of its findings. First, the utilization of the I-change model as an integrated framework offers a comprehensive approach to examining the motivational and behavioral factors associated with induced abortion. This model offers a solid foundation for analyzing various aspects of abortion-related decision-making and actions. Furthermore, the inclusion of men and conducting couple-based interviews is another strength of this study. By integrating the perspectives of both men and women, a more holistic understanding of the intricate complexities and dynamics surrounding induced abortion can be attained. This inclusive approach enables a comprehensive exploration of the experiences and viewpoints of individuals within intimate relationships, providing valuable insights into the social and relational aspects of abortion decision-making. However, it is important to acknowledge certain limitations of this study. One such limitation is the potential reluctance of couples to participate, stemming from concerns about legal repercussions and the sensitive nature of the topic. To mitigate this limitation, the researcher will prioritize establishing trust with the couples by providing a clear explanation of the research objectives and ensuring the utmost confidentiality of any shared information. Another limitation is linked to the couple-centered interview approach, as it may influence the perspectives and responses of couples when they are together. To address this limitation, couples will be offered the choice to participate in individual face-to-face interviews. This option will enable each partner to express their viewpoints independently, potentially yielding a more comprehensive understanding of their experiences and perspectives. By acknowledging these constraints and implementing appropriate measures to mitigate them, the study strives to minimize potential biases and augment the validity and reliability of the research outcomes.

## Data Availability

No datasets were generated or analysed during the current study.
